# Steering a Tractor by Means of an EMG-Based Human-Machine Interface

**DOI:** 10.3390/s110707110

**Published:** 2011-07-11

**Authors:** Jaime Gomez-Gil, Israel San-Jose-Gonzalez, Luis Fernando Nicolas-Alonso, Sergio Alonso-Garcia

**Affiliations:** Department of Signal Theory, Communications and Telematics Engineering, University of Valladolid, 47011 Valladolid, Spain; E-Mails: ijosgon@ribera.tel.uva.es (I.S.-J.-G.); lnicalo@ribera.tel.uva.es (L.F.N.-A.); salonsog@ribera.tel.uva.es (S.A.-G.)

**Keywords:** agricultural vehicles, human-machine interface (HMI), human-computer interface (HCI), brain-computer interface (BCI), electroencephalography (EEG), control, global positioning system (GPS), tractor, guidance

## Abstract

An electromiographic (EMG)-based human-machine interface (HMI) is a communication pathway between a human and a machine that operates by means of the acquisition and processing of EMG signals. This article explores the use of EMG-based HMIs in the steering of farm tractors. An EPOC, a low-cost human-computer interface (HCI) from the Emotiv Company, was employed. This device, by means of 14 saline sensors, measures and processes EMG and electroencephalographic (EEG) signals from the scalp of the driver. In our tests, the HMI took into account only the detection of four trained muscular events on the driver’s scalp: eyes looking to the right and jaw opened, eyes looking to the right and jaw closed, eyes looking to the left and jaw opened, and eyes looking to the left and jaw closed. The EMG-based HMI guidance was compared with manual guidance and with autonomous GPS guidance. A driver tested these three guidance systems along three different trajectories: a straight line, a step, and a circumference. The accuracy of the EMG-based HMI guidance was lower than the accuracy obtained by manual guidance, which was lower in turn than the accuracy obtained by the autonomous GPS guidance; the computed standard deviations of error to the desired trajectory in the straight line were 16 cm, 9 cm, and 4 cm, respectively. Since the standard deviation between the manual guidance and the EMG-based HMI guidance differed only 7 cm, and this difference is not relevant in agricultural steering, it can be concluded that it is possible to steer a tractor by an EMG-based HMI with almost the same accuracy as with manual steering.

## 1. Introduction

In recent years, research in agricultural vehicle guidance has been focused on autonomous tractor guidance, which has been mainly performed using a satellite-based Global Positioning System (GPS) [1–5]. Machine vision [6–9] and multiple sensors [10–13] are positioning methods that have also been employed to achieve autonomous guidance. Research in the teleoperation of tractors [14], the use of multiple autonomous robots [15], augmented reality, [16] and tractor architecture and communications [17] can also be found.

Scientific literature shows that the employment of human-computer interfaces (HCIs) and brain-computer interfaces (BCIs) has allowed some interesting advances in areas loosely related to tractor guidance. In the medical research area, HMI and BCI have been employed to allow people with disabilities to guide wheelchairs [18–20]. Vehicle guidance could benefit from the use of HMI and BCI, which allows for the prediction of voluntary human movement more than one-half second before it occurs [21–23], and allows for the detection of driver fatigue [24–26] and driver sleepiness [27–30].

This article explores the use of new interfaces in the agricultural field by employing an HMI to steer a tractor. To the best of our knowledge, no similar research has been reported in an agricultural scenario.

## 2. Electrical Signals on the Scalp Surface

The human nervous system is an organ system composed of the brain, the spinal cord, the retina, nerves and sensory neurons [31]. These elements produce electrical activity that can be measured in different ways and places. The measurement of this electrical activity in the scalp using noninvasive electrodes offers electromyography (EMG) signals related to muscle activation and electroencephalographic (EEG) signals related to brain activity.

The measurement of the EMG signals associated with a muscle’s activation is usually performed near it. But the high relative power of the EMG signals makes them propagate far from the muscles. EMG signals from the jaw, tongue, eye, face, arm and leg muscles can be measured on specific points of the scalp surface. The specific EMG signals corresponding to eye movements are named electrooculographic (EOG) signals.

EEG signals related to brain activity can also be measured on the scalp surface. Due to the high power of EMG signals, the measurement of the EEG signals is often contaminated by EMG artifacts, which EMG signals present in the EEG recordings. To achieve EEG signals without EMG artifacts: (i) the user must avoid moving muscles in the EEG signal acquisition; and (ii) some signal processing algorithms can be accomplished to remove EMG artifacts from the EEG signals acquired [32–34].

## 3. Surface EMG and EEG Signals Applied for Control

The acquisition and process of EMG signals from voluntary activated muscles offers a communication path that, for either disabled or healthy people, can be used in many tasks and in different environments. Some of these tasks applied for disabled people are the control of a robotic prosthesis [35–37] or a wheelchair [38–40], as well as computer [41–43] or machine [44–46] interaction. The interfaces for games [47–49] and virtual reality [50–52] are environments where healthy people can communicate through EMG signals.

In contrast to the acquisition and process of EMG signals from voluntarily activated muscles, the acquisition and process of EEG signals is focused for people with severe disabilities that lose all voluntary muscle control, including eye movements and respiration. In this way, robotic prosthesis [53–55] or wheelchair control [56–58] are also tasks in which EEG-based interfaces can be useful. Moreover, healthy people can also employ EEG-based interface environments such as, again, interfaces for games [59–61] and virtual reality [62–64].

EMG computer interface [65], human-computer interface (HCI) [66], EMG-based human-computer interface [67], EMG-based human-robot interface [68], muscle-computer interface (MuCI) [69], man-machine interface (MMI) [70], and biocontroller interface [71] are different terms used in the scientific literature to name communication interfaces that can employ EMG signals, among others. In contrast, the widely accepted name for brain communication through exclusively EEG signals that are independent of peripheral nerves and muscles is brain-computer interface (BCI).

**Figure 1.**
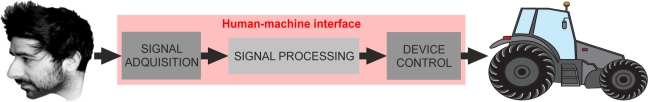
Block diagram of the application of a human-machine interface applied into a tractor steering.

The block diagram of an EMG-based HMI or a BCI applied to control a machine usually comprises three blocks: a s*ignal acquisition* block where EMG or EEG signals are acquired from the user by means of electrodes, a *signal processing* block where the signals acquired are processed to obtain information about the user status, and a *device control* block that acts on the machine (Figure 1). The EMG or EEG signal acquisition can be done with electrodes placed on the body or scalp of the user, or with electrodes placed inside the body, being these acquisitions referred to as non-invasive or invasive, respectively. The statistical analysis [72], Bayesian approaches [73], neural networks [74], time frequency procedures [75], and parametric modeling [76] are usual techniques employed in the *signal processing* block, estimating the user status from the acquired EMG or EEG signals. This is the most complex part of EMG-based HCIs or BCIs, because it needs to process jointly the signals acquired from all electrodes. Furthermore, each electrode signal is composed in turn by the sum of a large number of signals at the same and at different frequencies, which comes to each electrode from different parts of the user body or brain. On-off switch [77], proportional-integral-derivative [78], and fuzzy logic [79] control are control types usually employed in the *device control* block.

## 4. Materials and Methods

### 4.1. The Emotiv EPOC Interface

The EPOC is a low cost Human-Computer Interface (HCI) that is comprised of: (i) a neuroheadset hardware device to acquire and preprocess EEG and EMG user brainwaves, and (ii) the software development kit (SDK) to process and interpret these signals. It can be purchased from the Emotiv Company website for less than one thousand US dollars [80].

The neuroheadset acquires brain neuro-signals with 14 saline sensors placed on the user scalp. It also integrates two internal gyroscopes to provide user head position information. The communication of this device with a PC occurs wirelessly by means of a USB receiver.

Emotiv provides software in two ways: (i) some suites, or developed applications, with graphical interface to process brain signals, to train the system, and to test the neuroheadset; and (ii) an application programing interface (API) to allow users to develop C or C++ software to be used with the neuroheadset.

The Emotiv EPOC can capture and process brainwaves in the Delta (0.5–4 Hz), Theta (4–8 Hz), Alpha (8–14 Hz), and Beta (14–26 Hz) bands. With the information from signals in these bands, it can detect expressive actions, affective emotions, and cognitive actions.

The expressive actions correspond to face movements. Most movements have to be initially trained by the user, and as the user supplies more training data, the accuracy in the detection of these actions typically improves. The eye and eyelid-related expressions *blink*, *wink*, *look left*, and *look right* cannot be trained because information about these expressions relies on the Emotiv software.

The affective emotions detectable by the Emotiv EPOC are *engagement*, *instantaneous excitement*, and *long-term excitement*. None of these three has to be trained.

Finally, the Emotiv EPOC works with 13 different cognitive actions: the *push*, *pull*, *left*, *right*, *up* and *down* directional movements, the *clockwise*, *counter-clockwise*, *left*, *right*, *forward* and *backward* rotations and a special action that makes an object disappear in the user mind.

**Figure 2.**
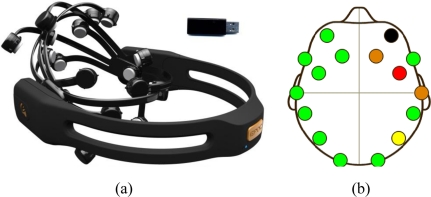
(**a**) The Emotiv EPOC neuroheadset and the wireless USB receiver. (**b**) A picture that shows with intuitive colors the contact quality of the neuroheadset on the user head.

Figure 2(a) shows an Emotiv EPOC neuroheadset photograph, and Figure 2(b) shows with intuitive colors the contact quality of the neuroheadset on the user head. This picture was screen-captured from a software application provided by Emotiv.

### 4.2. Hardware of the Developed System

**Figure 3.**
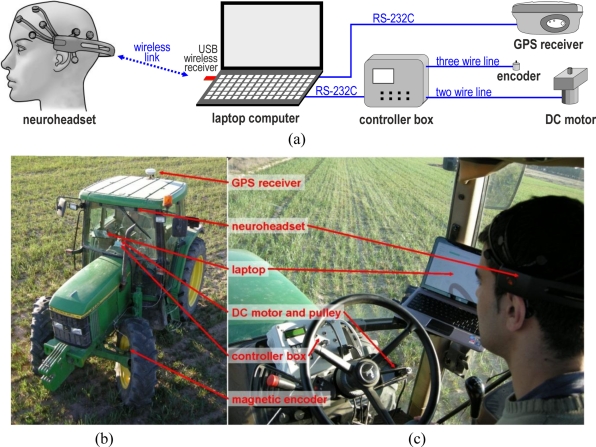
(**a**) Schematic of the connections between the hardware components of the developed system. (**b**) Tractor used in the tests. (**c**) Photo of the driver inside the tractor.

Figure 3(a) shows the hardware components of the system and the connections between them. All components were mounted on a 6400 John Deere tractor (Figure 3(b,c)). As mentioned, the HMI model was an EPOC, from the Emotiv Company [80].

A DC RE-30 Maxon motor was installed to move the steering wheel by means of a reducer gear and a striated pulley. A controller box was specially designed to steer the tractor continuously according to the commanded orders sent by the laptop [81]. To achieve the desired angle, the box uses fuzzy logic control technology to power the DC motor by means of a PWM signal. This controller box measures the steering angle with a magnetic encoder.

An R4 Trimble receiver was used to measure the real trajectories of the results section and to perform the autonomous GPS guidance. The update of positions was configured to a rate of 5 Hz. This receiver employed real time kinematic (RTK) corrections to achieve an estimated precision of 2 cm. The corrections were provided by a virtual reference station (VRS) managed by the ITACyL, a Spanish regional agrarian institute.

A laptop computer ran our developed application, which was continuously: (i) obtaining information from the BCI about the driver brain activity, (ii) sending steering commands to the controller box about the desired steering angle, and (iii) saving the followed trajectory, obtained from the GPS.

### 4.3. Software of the Developed System

**Figure 4.**
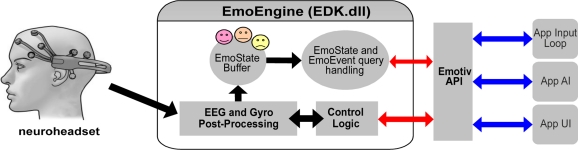
Diagram of the integration of the EmoEngine and the Emotiv API with an application.

The Emotiv EPOC includes the Emotiv API, a C++ API, which allows communication with the Emotiv headset, reception of preprocessed EEG/EMG and gyroscope data, management of user-specific or application-specific settings, post-processing performing, and translation of the detected results into an easy-to-use structure called EmoState. The EmoEngine is the logical abstraction of the Emotiv API that performs all the processing of the data from the Emotiv headset. The EmoEngine is provided in a edk.dll file, and its block diagram is shown in Figure 4.

The Emotiv EPOC, by means of the Emotiv API, provides to external applications information about the event type that the device estimates emanates from the user brain and reports the event power, which represents the certainty of the event estimation. A neutral event is reported when no actions are detected.

**Figure 5.**
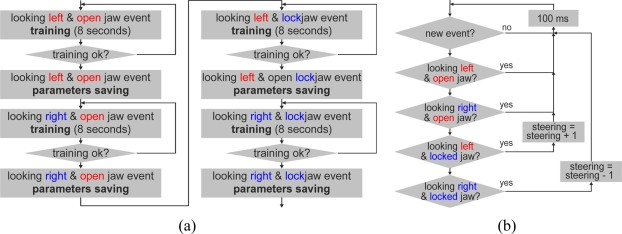
Simplified flow chart of the (**a**) system training of the four events that the BCI has to detect and (**b**) system test following a trajectory with the tractor.

A C++ application was developed to receive the processed user brain information through the Emotiv API and to steer the tractor through the controller box. This application was configured to train some events according to the flow chart of Figure 5(a), and then, according to the flow chart of Figure 5(b), to operate with these events to steer the tractor. The chosen events in the developed application were four combinations of muscle movements:

- the user eyes looking to the left when the user’s jaw is open;
- the user eyes looking to the right when the user’s jaw is open;
- the user eyes looking to the left when the user’s jaw is closed;
- the user eyes looking to the right when the user’s jaw is closed.

The test driver was trained to use these events. In the training process, the EmoEngine analyzes the driver brainwaves to achieve a personalized signature of each particular event as well as one of a neutral background state. These signatures are stored in the EmoEngine memory. In the tractor steering process, the EmoEngine analyzes in real time the brainwaves acquired to detect signatures that match one of the previously stored signatures in the EmoEngine memory, and when this occurs, it communicates to the application that a specific event with a specific power emanated from the user brain.

### 4.4. Methods

The steering using the Emotiv EPOC was compared with the two usual methods of tractor steering: manual steering and autonomous GPS steering. A healthy driver tested the tractor guidance manually and through the Emotiv EPOC interface. The Emotiv EPOC training was completed by the driver before testing the system with the tractor. The guidance speed to test the system was approximately 1 m/s. The 5 Hz GPS rate allowed acquiring positions in the tractor trajectories approximately 20 cm apart.

The trajectories where this comparison was accomplished were: (i) a straight line longer than 50 m; (ii) a 10 m step; and (iii) a circumference of 15 m radius. These three trajectories were drawn over the plot with a mattock, taking into account GPS reference points, in order that the driver testers could follow the trajectories in the tests of manual guidance and in the tests performed through the Emotiv EPOC Interface. These three trajectories were programed with the computer for the autonomous GPS guidance tests.

The control law of Equation (1) was employed in the automatic GPS guidance. In this equation *δ* is the steering angle, *x* is the distance of the tractor from the desired trajectory, *θ* is the difference between the tractor orientation and the reference trajectory orientation in the trajectory point nearest to the tractor, *L* is the distance between the tractor axles, and *k_1_, k_2_* are the control gains [3,10,13]:

(1) $$\delta =\arctan(({-k}_{1}\cdot x-k_{2}\cdot \tan\;\theta )L\cdot \cos^{3}\theta )$$

The four muscle events enumerated in the *Software of the Developed System* section were initially trained with the driver who tested the system. Later, these events were used to perform the guidance through HMI along the three different trajectories. When the driver failed to follow the desired trajectory by EMG-based HMI guidance because he was not completely attentive, another attempt was performed. The authors’ initial intention was to train and use only the first two events, but we noticed that the trained events were detected during real tests in the HMI system when the driver only looked to the right or to the left, independently of the jaw status. To provide the system more information about jaw status, it was necessary to train and use all four events instead of only two.

## 5. Results and Discussion

### 5.1. Experimental Results

**Figure 6.**
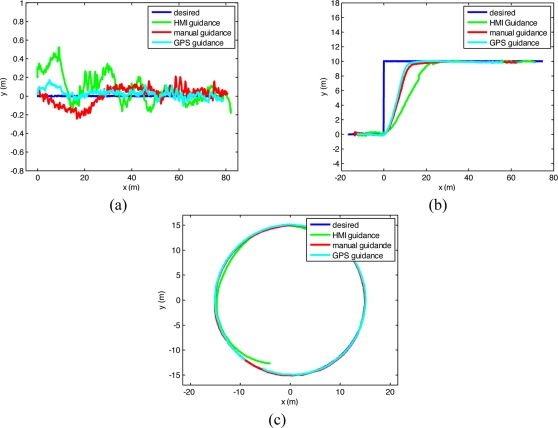
Real test guidance results through the HMI, with manual guidance, and with automatic GPS steering, taking as desired trajectories (**a**) a straight line, (**b**) a step and (**c**) a circumference.

Real tests were accomplished in Pozal de Gallinas, Spain, in March 2011, along the trajectories and with the procedures presented in the Methods section. The autonomous guidance control law of Equation (1) was experimentally tuned, and k_1_ = 0.1 and k_2_ = 0.35 were obtained. Figure 6 shows the obtained results along the three trajectories. Table 1 presents the mean, standard deviation, and range of the distance from the performed trajectory by the tractor to the 50 m straight desired trajectory (Figure 6(a)). The step trajectory of Figure 6(b) was considered as a step input to the system for obtaining the step response. Table 2 presents the settling distance produced by the 10 m step response in the system. The settling distance is the horizontal distance that the tractor needed to advance after the 10 m step for the tractor to be in ±5% of the step size from the final desired trajectory, that is, to be between ±0.5 m from the final desired trajectory. Table 3 presents the mean, standard deviation, and range of the distance from the performed trajectory to the 15 m radius circular desired trajectory (Figure 6(c)).

As it can be perceived from the trajectories of and from the data of Tables 1, 2 and 3, the guidance accuracy through the HMI was lower than that obtained when the driver employed his hands, and this was lower than that obtained by the autonomous GPS guidance.

### 5.2. Discussion

The tests comparing the HMI guidance with the manual or autonomous GPS tractor guidance show that the EMG-based HMI guidance system: (i) offers lower accuracy, because the precision achieved with the HMI was lower than that obtained with manual steering, which was also below that obtained by the autonomous GPS tractor guidance; (ii) requires extra training time, because the guidance through the HMI required a lengthy training process; and (iii) requires higher user concentration, because the drivers employing the HMI needed to be very focused to follow the desired trajectories successfully. Therefore, the authors consider that at present, vehicle guidance through EMG-based HMIs might be of interest only for people with disabilities who cannot manage a steering wheel by hand. Nevertheless, the EMG-based HMI guidance offers reasonably good accuracy, with only 16 cm standard deviation of error, which is acceptable for most agricultural tasks, and is not very different from the 9 and 4 cm obtained by means of manual steering and automatic GPS steering, respectively.

The Emotiv Company declares that the EPOC device acquires and processes EEG signals [80], and therefore, is a BCI. Moreover, most scientific literature considers the Emotiv EPOC as a BCI [59,82–89]. A BCI is a direct communication between the brain and a computer. This communication is based on the capture and process of EEG signals of brain activity and is independent of nerve and muscle activity. In turn, HCI and HMI are communications methods that encompass a wide variety of mechanisms, including the acquisition and processing of EMG signals associated to muscle movements. In our research, guidance tests by means of the Emotiv EPOC interface were unsuccessful when the Emotiv EPOC training and testing did not imply muscle movements, which means, when the drivers have only the cognition but have not performed the movements, steering was not possible. Therefore, the authors trained and employed events related with eye and jaw muscle movements, which were better detected by the EPOC. For this reason, the authors consider that the Emotiv EPOC is an HCI that proceses mainly EMG signals of muscle movements, but not a real BCI that only proceses EEG brainwaves. Moreover, since the tests performed with this HCI device is applied to a machine, the authors refer to the developed EPOC system as an EMG-based HMI.

The steering of vehicles by means of devices such as steering wheels or joysticks need to update the steering wheel or the joystick positions approximately every second. This steering can be performed by EMG-based HMIs, as this article proves. The actual BCI technology can only update the steering wheel or the joystick position at rates lower than 0.5 Hz, because the mean time to transmit a command is greater than 2 s [90–95]. Therefore, the vehicle guidance by means of BCI technology is usually achieved in the research literature by just choosing destinations from a list or selecting the branch in each intersection of the possible paths [56,57,96]. After the selections of the destination by means of the BCI, a completely autonomous guidance system steers the vehicle without user intervention. In summary, an EMG-based HMI guidance system allows for continuously updating the steering, but this updating is hard to perform through BCIs because the time to transmit a command by BCIs is greater than 2 s. One limitation of the EMG-based HMI tractor guidance is that the drivers need to be completely focused to follow the desired trajectory successfully.

The EMG-based HMI presented may be useful in practice compared to standard manual control for people with physical disabilities. Comparing the EMG-based HMI presented by other interfaces for people with disabilities based on mechanical sensors that measure movements in the user body produced by healthy muscles, the proposed system could offer three advantages. First, an easier installation and removal, because it is simpler to don and doff a helmet than install a mechanical sensor on some body parts. Second, a simpler calibration, because it could be simpler to train movements by the Emotiv EPOC software than to calibrate specific sensors attached to the driver’s body. Third, a lower price, because the Emotiv EPOC is a general purpose device, and this allows the Emotiv EPOC hardware to be purchased for less than $500, whereas specific purpose acquisition and installation of sensors on the body of the user would probably surpass this cost.

Moreover, future lines of research with tractors steering through HMIs that integrate both EMG and EEG signals could provide additional advantages over conventional guidance. One possible advantage may be the capability of this system to detect fatigue [24–26] or sleepiness [27–29] from the EEG signals, and to employ this information for evaluating the concentration of the driver and for suggesting necessary breaks. In this way, safer farm work might be achieved. Another line of research may be if HMIs could detect in advance, with regard to muscle movement, some special situations where the tractor needs to be immediately stopped. Research literature indicates that voluntary human movements can be predicted more than one-half second before they occur [21–23]. This advantage may also contribute to safer farm work. Finally, future research will also have to show if the BCI communication could allow people with severe physical disabilities to steer tractors only by thinking.

## 6. Conclusions

In summary, it is possible to steer a tractor through an EMG-based HMI. In comparison with manual or automatic GPS guidance, the accuracy was lower in the EMG-based HMI. Nevertheless, since the difference between the standard deviation of error to the desired trajectory in the real test between EMG-based HMI guidance and manual steering was only a few centimeters, and this difference is not relevant for most agricultural tasks, it can be concluded that is possible to steer a tractor by an EMG-based HMI with almost the same accuracy as with manual steering.

## Figures and Tables

**Table 1.** Mean, standard deviation, and range of the distance from the performed trajectory to the desired trajectory in the 50 m straight line.

	**GPS guidance**	**Manual guidance**	**HMI guidance**

**Mean (cm)**	1.2	2.9	10.6
**Standard deviation (cm)**	4.2	8.7	15.8
**Range (cm)**	0–17.2	0–24.3	0–52.3

**Table 2.** Settling distances for the 10 m step reference trajectory.

	**GPS guidance**	**Manual guidance**	**HMI guidance**

**Settling distance (m)**	13.3	14.3	23.1

**Table 3.** Mean, standard deviation, and range of the distance from the performed trajectory to the desired trajectory in the 15 m radius circumference.

	**GPS guidance**	**Manual guidance**	**HMI guidance**

**Mean (cm)**	1.9	3.9	13.7
**Standard deviation (cm)**	6.6	11.2	26.6
**Range (cm)**	0–25.0	0–27.6	0–74.5
